# Design and Sound Absorption Characteristics of a Broadband Acoustic Absorber for Transformer Noise Control Based on a Composite Structure of Traditional and Side-Slit Helmholtz Resonators

**DOI:** 10.3390/ma19163380

**Published:** 2026-08-08

**Authors:** Lei Zhao, Guanghui Yang

**Affiliations:** School of Mechanical Engineering, University of Science and Technology Beijing, Beijing 100083, China; 15266572564@163.com

**Keywords:** transformer noise, side-slit Helmholtz resonator, acoustic metamaterial, composite structure, broadband sound absorption

## Abstract

This study proposes a targeted sound-absorption design strategy to address the challenge of broadband and low-frequency transformer noise. Based on field measurements, transformer noise is identified as being characterized by a dominant 100 Hz fundamental component, pronounced low-order harmonics in the range of 200–500 Hz, and an overall broadband distribution within 100–1000 Hz. To meet these noise-control requirements, a composite acoustic metamaterial based on a traditional Helmholtz resonator and a side-slit Helmholtz resonator (HRSS-HR) is proposed. An electro-acoustic analogy model is established to predict its acoustic performance and is validated through finite element simulation and impedance tube experiments, thereby revealing the underlying sound-absorption mechanisms. The proposed composite unit cell exhibits four characteristic absorption peaks over a broad frequency range, among which the peaks within 100–1000 Hz are directly relevant to transformer noise control. We systematically investigate the evolution of sound absorption from a single unit to coupled parallel structures and then to a 4 × 4 array, leveraging the tunability of the HRSS-HR structure. Parallel coupling broadens the effective absorption bandwidth but weakens the original impedance matching condition, whereas increasing the slit width and decreasing the neck height can effectively recover the low- and middle-frequency absorption performance. Finally, a 4 × 4 HRSS-HR composite metamaterial array is developed. Both simulation and experiment confirm that the optimized array maintains relatively high absorption levels near 100 Hz and the major harmonic bands of 200–500 Hz, while preserving good broadband absorption capability over 100–1000 Hz. This work validates the strong application potential of the proposed HRSS-HR design in transformer broadband noise control and offers a practical solution for low-frequency harmonic noise mitigation in transformers and similar power equipment.

## 1. Introduction

With the rapid growth of urbanization and the increasing proximity of substations to residential, commercial, and industrial areas, transformer noise has become an increasingly prominent environmental and engineering issue. In practical operation, transformer noise has become a prominent environmental and engineering concern, and is typically characterized by harmonic components with 100 Hz as the fundamental frequency [1,2,3]. In addition, the cooling system introduces further mid-frequency broadband components, making transformer noise a coupled acoustic field with both tonal low-frequency harmonics and broadband characteristics. Under increasingly stringent requirements for environmental noise control, it is therefore necessary to develop efficient and compact sound-absorbing structures specifically for transformer noise mitigation.

Current transformer noise control strategies mainly rely on source optimization, sound barriers, enclosures, vibration isolation, and conventional sound-absorbing materials. However, these methods still suffer from clear limitations in the low-frequency range. Conventional sound-insulating structures are strongly constrained by the mass law and generally require large structural mass or thickness to achieve effective low-frequency attenuation. Similarly, traditional porous or fibrous absorbers usually require considerable thickness to obtain high absorption efficiency at low frequencies, which conflicts with the practical requirements of compactness, heat dissipation, maintenance accessibility, and engineering adaptability in transformer-related applications [4,5,6,7,8]. These limitations have motivated the search for novel acoustic structures capable of achieving broadband and low-frequency absorption within a subwavelength thickness.

In recent years, acoustic metamaterials have provided a promising route for overcoming these limitations. Through local resonance, coiled-channel propagation [8,9,10,11,12], Helmholtz resonance, and hybrid coupling mechanisms, acoustic metamaterials can realize low-frequency sound absorption with compact dimensions. Among these structures, Helmholtz-resonator-based acoustic metamaterials have attracted particular attention due to their compact geometry and flexible parameter tunability [13,14,15,16]. A large number of studies have shown that the acoustic performance of Helmholtz resonators can be effectively tailored by adjusting neck dimensions, cavity geometry, and coupling topology. To broaden the effective absorption bandwidth, researchers have proposed gradient structural designs, multi-cavity coupling, nested resonators, and hybrid structures combined with coiled or labyrinthine channels [17,18,19,20,21]. Nevertheless, despite these advances, conventional Helmholtz-based metamaterials still commonly suffer from narrow absorption bandwidths, incomplete inter-peak coupling, or excessive structural complexity, which limit their engineering applicability.

For transformer noise control, the above limitations are particularly critical. Unlike general low-frequency noise mitigation, transformer noise control requires strong absorption at the 100 Hz fundamental frequency. It also demands effective suppression of the major low-order harmonics (200–500 Hz) and satisfactory performance across the 100–1000 Hz broadband range. Therefore, the design objective is not simply to obtain a single strong absorption peak, but to construct a compact absorber capable of targeted multi-peak and broadband sound absorption. In this context, the side-slit Helmholtz resonator [22] provides an effective means of introducing multiple resonant modes through slit-related acoustic pathways, while the traditional Helmholtz resonator offers strong tunability for low-frequency resonance. A rational combination of these two resonant elements is therefore expected to provide a feasible route toward transformer-oriented broadband acoustic absorption.

To address this problem, we propose a composite acoustic metamaterial that combines a traditional Helmholtz resonator (HR) and a side-slit Helmholtz resonator (HRSS) for transformer noise control. Based on field measurements, the target control bands are first identified as the 100 Hz fundamental frequency, the major harmonics in the range of 200–500 Hz, and the overall 100–1000 Hz broadband band. On this basis, an HRSS-HR composite unit cell is constructed, and an electro-acoustic analogy model is established to predict its acoustic performance. The theoretical results are further verified by finite element simulations and impedance tube experiments to clarify the underlying sound-absorption mechanisms. We also systematically investigate the evolution of sound absorption from a single unit to coupled parallel structures and then to a 4 × 4 array.

The remainder of this paper is organized as follows. Section 2 identifies the target control frequency bands for transformer noise based on field measurements. Section 3 presents the design concept of the proposed composite acoustic metamaterial and establishes the corresponding theoretical and numerical models. Section 4 investigates the sound absorption behavior of the optimized composite unit and the 4 × 4 array, including the effects of key structural parameters, coupling characteristics, and experimental verification. Finally, Section 5 summarizes the main conclusions of this work.

## 2. Target Frequency Identification for Transformer Noise Control

### 2.1. Measurement Setup and Positions

**Figure 1:**
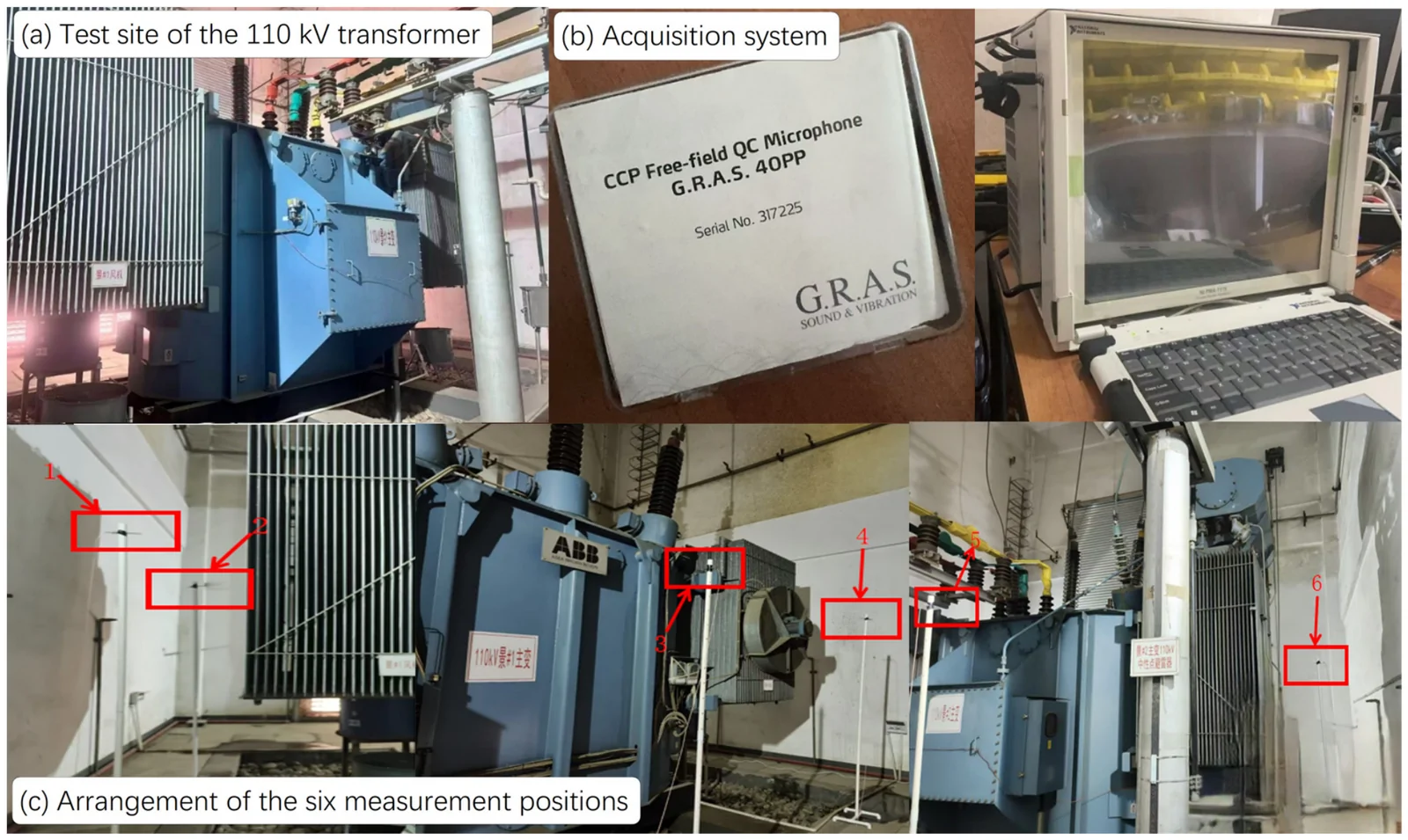
Field measurement setup for transformer noise identification: (**a**) Test site of the 110 kV transformer; (**b**) acquisition system; (**c**) arrangement of the six measurement positions.

Field measurements were first conducted at a 110 kV substation to establish a practical design target. Figure 1 illustrates the field measurement setup, including the test site, the acquisition system, and the arrangement of the six measurement positions around the transformer. The measurement positions were arranged around the transformer body, radiator surfaces, and fan side in order to distinguish the contributions of structural radiation noise and cooling-system noise [23,24]. According to the measurement scheme, all points were positioned 1 m away from the major radiation surfaces and 2 m above the ground. The ambient background noise during the test was 53.1 dBA, more than 12 dBA below the transformer operating noise, satisfying the requirement for reliable acoustic analysis. The acquisition system comprised an NI cDAQ-9188 platform and G.R.A.S. 40PP free-field microphones to capture sound pressure signals over the frequency range relevant to transformer noise control.

### 2.2. Spectral Characteristics of Transformer Noise

**Figure 2:**
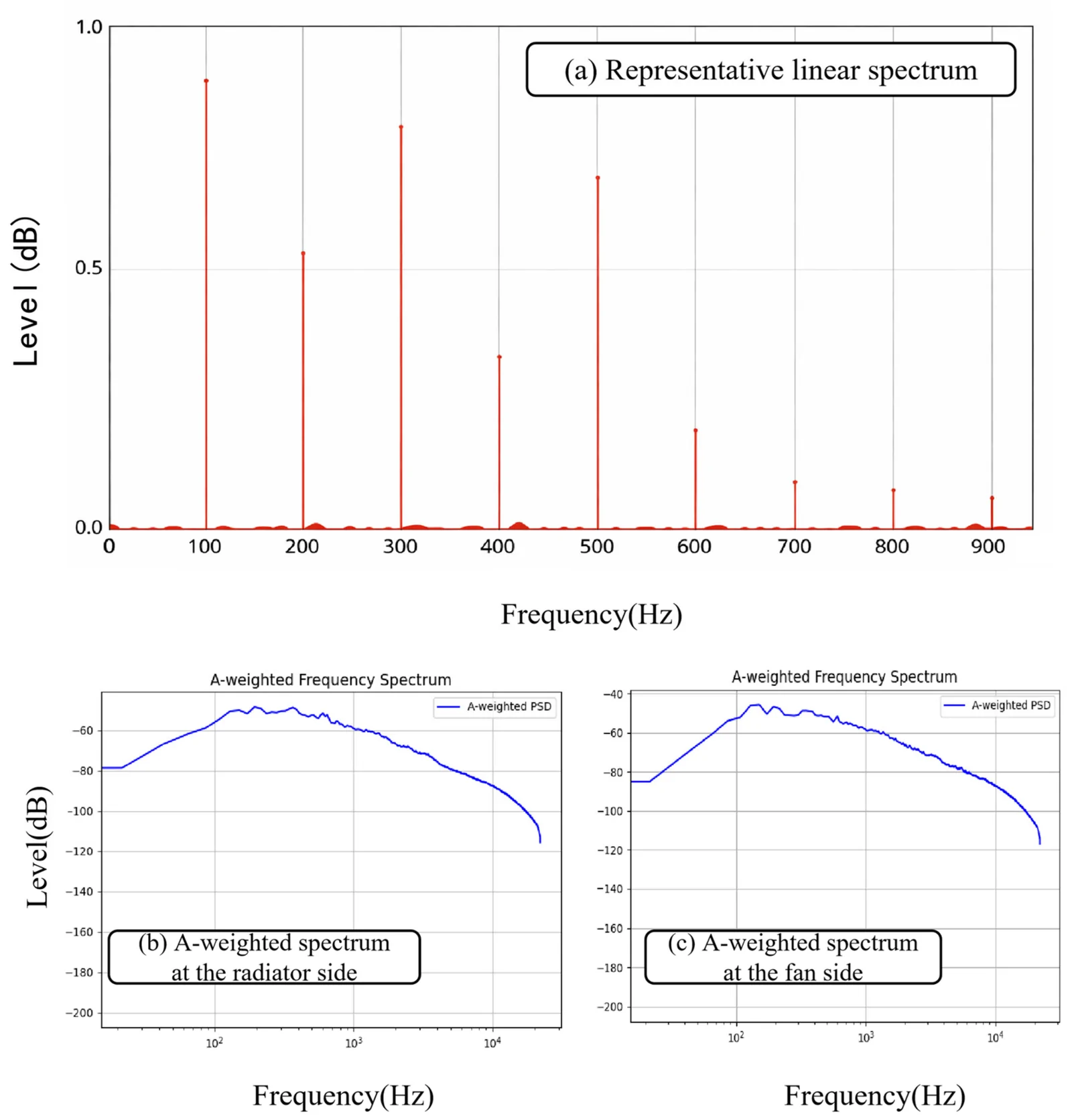
Measured spectral characteristics of transformer noise: (**a**) representative linear sound pressure level spectrum at P1; (**b**) A-weighted spectrum at P1; (**c**) A-weighted spectrum at P4.

The measured spectra show that transformer noise exhibits typical low-frequency-dominated characteristics with pronounced harmonics. In the linear sound pressure level spectrum, the 100 Hz fundamental frequency and the harmonics at 200, 300, 400, and 500 Hz are significantly higher than the surrounding components, indicating that electromagnetic vibration of the transformer core is the major source of acoustic radiation. As shown in Figure 2a, the dominant tonal components are clearly concentrated at 100 Hz and its low-order harmonics.

After A-weighting, the low-frequency components are partially attenuated in perceived loudness, whereas the frequency range from 100 to 1000 Hz still remains prominent because it contains both the low-order harmonic contribution and the medium-frequency contribution associated with the cooling system. As shown in Figure 2b,c, the A-weighted spectra at the radiator side and the fan side both indicate that the 100–1000 Hz range remains acoustically important, although the relative spectral distributions differ because of the different dominant noise sources at these positions.

These results indicate that transformer noise is essentially a coupled acoustic field consisting of strong low-frequency tonal components and broadband medium-frequency content. Therefore, the target control bands of the present study are identified as the 100 Hz fundamental frequency, the major low-order harmonics in the range of 200–500 Hz, and the overall broadband range of 100–1000 Hz. This target-frequency identification provides the practical basis for the subsequent design of the proposed broadband acoustic metamaterial.

## 3. Composite Acoustic Metamaterial

**Figure 3:**
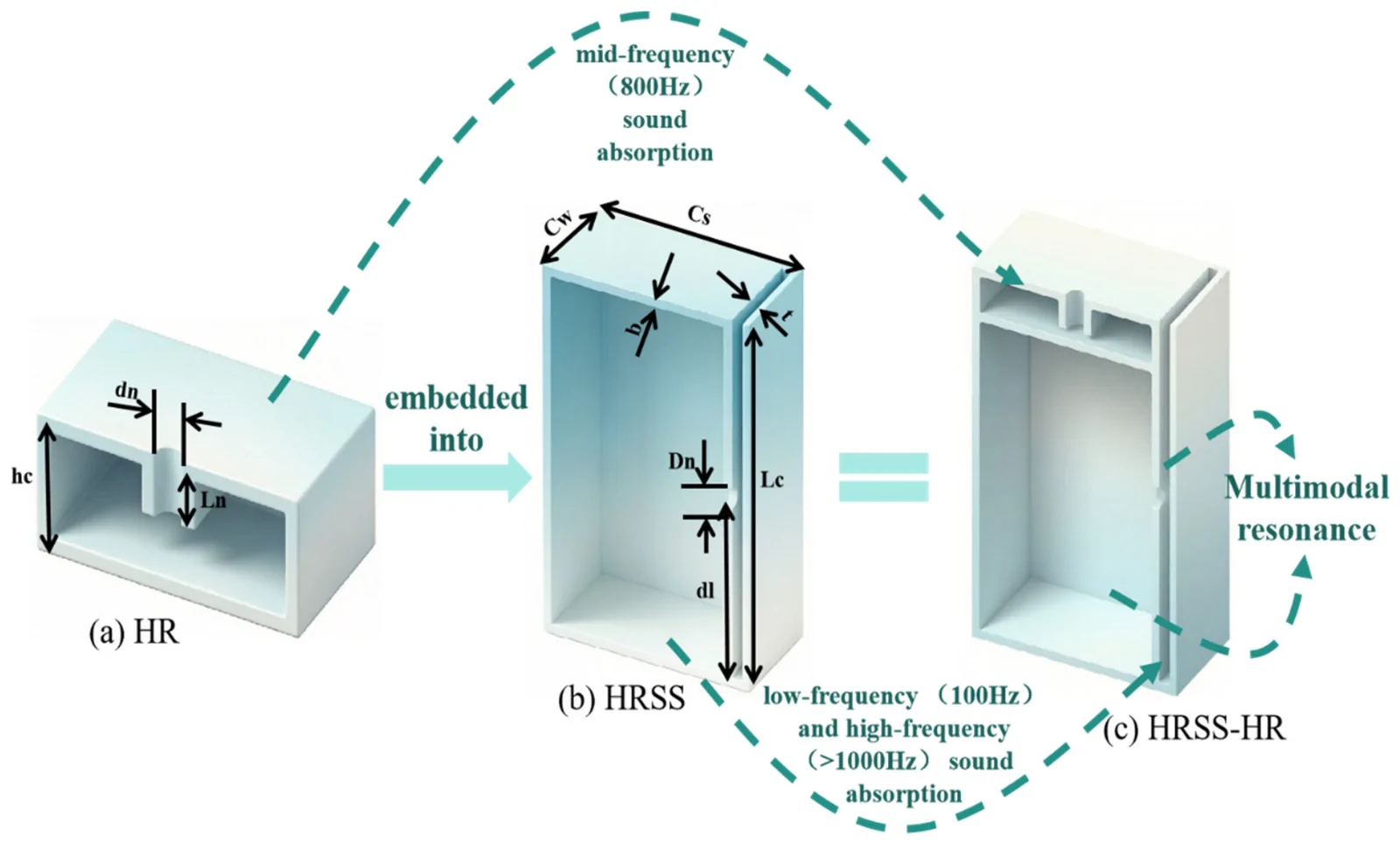
Schematic diagram of the proposed side-slit Helmholtz resonator–Helmholtz resonator composite structure: (**a**) traditional HR unit; (**b**) side-slit HRSS unit; (**c**) proposed composite HRSS-HR unit.

The schematic diagram of the proposed HRSS-HR composite structure is shown in Figure 3. The structure is constructed by embedding a traditional Helmholtz resonator (HR) unit (Figure 3a) into a side-slit Helmholtz resonator (HRSS) unit (Figure 3b), thereby forming an HRSS-HR composite unit cell (Figure 3c). The HRSS unit provides low- and high-frequency absorption peaks through multimode resonance induced by coupling between the side-slit waveguide and the Helmholtz-type resonance path, while the embedded HR unit introduces an additional absorption peak in the middle-frequency range. Through this composite design, the proposed structure exhibits multiple characteristic absorption peaks over a broad frequency range, thereby improving the broadband sound absorption performance for transformer noise control. The key geometric parameters of the HRSS unit include cavity width *Cw*, cavity depth *Cs*, slit length *Lc*, slit width *t*, wall thickness *b*, perforation-related dimension *dl*, and perforation diameter *Dn*, whereas those of the HR unit include neck diameter *dn*, neck height *Ln*, and cavity height *hc*.

### 3.1. Theoretical Method

The sound absorption coefficient α is defined as the ratio of absorbed sound energy to incident sound energy, and for a rigidly backed absorber under normal incidence, it can be expressed as:

(1) $$a=1-{\left|\frac{Z_{s}-Z_{0}}{Z_{s}+Z_{0}}\right|}^{2}$$

where $Z_{s}$ is the specific surface acoustic impedance of the absorber and $Z_{0}=\rho_{0}c_{0}$ is the characteristic impedance of air, with $\rho_{0}$ and $c_{0}$ being the density and sound speed of air, respectively. Therefore, the key to the theoretical analysis is to determine the total surface acoustic impedance of the proposed HRSS-HR composite unit cell. This formulation follows the general modeling route adopted in previous studies on HRSS-based and resonator-coupled acoustic metamaterials [25].

Since the proposed composite unit cell is formed by embedding a traditional Helmholtz resonator (HR) unit into a side-slit Helmholtz resonator (HRSS) unit, its total impedance can be described as the parallel combination of the impedances of the two acoustic branches,

(2) $$\frac{1}{Z_{s}}=\frac{1}{Z_{HRSS}}+\frac{1}{Z_{HR}}$$

where $Z_{HRSS}$ and $Z_{HR}$ denote the specific surface impedances of the HRSS unit and the embedded HR unit, respectively.

**Figure 4:**
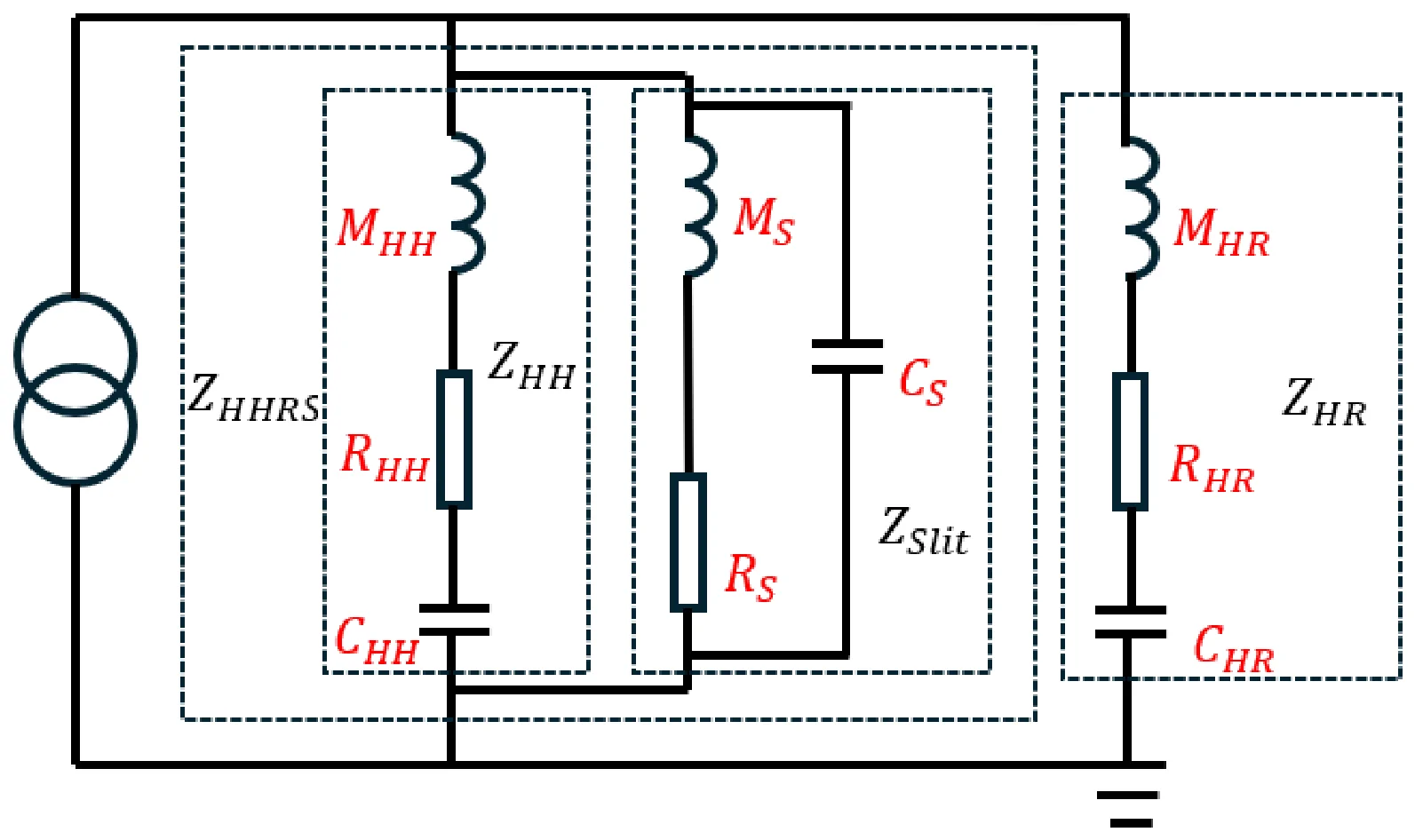
Equivalent circuit model of the HRSS-HR composite unit cell.

As shown in Figure 4, the equivalent circuit model of the proposed HRSS-HR composite unit cell exhibits a two-level parallel architecture, which forms the physical core for achieving broadband sound absorption. At the upper level, the total acoustic response is determined by the parallel coupling between the HRSS branch and the embedded HR branch. Within the HRSS branch, a secondary parallel coupling is introduced to accurately describe the cooperative interaction between the Helmholtz-type resonance path and the slit-waveguide resonance path. Specifically, the Helmholtz-type branch is represented by the series connection of $M_{HH}$, $R_{HH}$ and $C_{HH}$, whereas the slit branch is modeled by the parallel combination of $\left(M_{s}+R_{s}\right)$ and $C_{s}$.

The key feature of the present model lies in the parallel resonant structure of the slit branch, i.e., $\left(M_{s}+R_{s}\right)\cup C_{s}$. Unlike the conventional series resonance of a classical Helmholtz resonator, where the acoustic impedance reaches a minimum at resonance, the slit branch exhibits a maximum impedance $Z_{Slit}$ near its resonant frequency because the inductive reactance $j\omega M_{s}$ is cancelled by the capacitive reactance $1/j\omega C_{s}$. Under this condition, the incident acoustic wave is effectively blocked from entering the slit path and is instead redirected to strongly excite the parallel Helmholtz-type branch $Z_{HH}$, thereby generating an additional high-efficiency absorption peak within the target frequency band.

Thus, the broadband multi-peak absorption originates from the decoupled yet cooperative interaction between the Helmholtz resonance (series) and slit-waveguide resonance (parallel) within the parallel-coupled architecture. This equivalent circuit model also provides a solid theoretical basis for the subsequent targeted design and parameter optimization of the proposed structure.

For the HRSS branch, the transfer matrix method (TMM) is employed. According to the side-slit resonator model, the sound pressure and volume velocity at the inlet and rigidly backed end of the slit can be related by [26]

(3) $${\left[\begin{array}{c} p\\ u \end{array}\right]}_{x=0}=T_{HRSS}{\left[\begin{array}{c} p\\ u \end{array}\right]}_{x=Lc}=T_{A}T_{B}T_{C}{\left[\begin{array}{c} p\\ u \end{array}\right]}_{x=Lc}$$

where $T_{A}$, $T_{B}$, and $T_{C}$ are the transfer matrices corresponding to the left slit segment, the local resonant branch region, and the right slit segment, respectively. This decomposition is consistent with the HRSS modeling strategy reported in the literature.

The transfer matrix of a narrow slit segment can be written as [27]

(4) $$T_{Slit}=\left[\begin{array}{cc} cos\left(k_{f}l\right) & jZ_{f}sin\left(k_{f}l\right)\\ jsin\left(k_{f}l\right)/Z_{f} & cos\left(k_{f}l\right) \end{array}\right]$$

where *l* is the corresponding slit length, $k_{f}=\omega /c_{f}$ is the effective complex wave number, and $Z_{f}=\sqrt{K_{f}\rho_{f}}/S_{f}$ is the normalized acoustic impedance of the slit. Here, $S_{f}$ is the slit cross-sectional area, $c_{f}=\sqrt{K_{f}\rho_{f}}$, and $\rho_{f}$ and $K_{f}$ are the effective complex density and bulk modulus of air in the narrow slit, respectively, which are introduced to account for thermo-viscous losses.

The transfer matrix of the locally resonant region is expressed as [27]

(5) $$T_{B}=\left[\begin{array}{cc} 1 & 0\\ 1/Z_{h} & 1 \end{array}\right]$$

where $Z_{h}$ is the acoustic impedance of the Helmholtz-type side-branch resonator associated with the perforation and the adjacent cavity. In lumped form, $Z_{h}$ can be written as

(6) $$Z_{h}=R_{h}+j\omega M_{h}+\frac{1}{j\omega C_{h}}$$

where $R_{h}$, $M_{h}$, and $C_{h}$ denote the acoustic resistance, acoustic mass, and acoustic compliance of the side-branch resonator, respectively. Their expressions can be further written as

(7) $$M_{h}=\frac{\rho_{0}l_{eff,h}}{S_{h}},\;C_{h}=\frac{V_{h}}{\rho_{0}c_{0}^{2}}$$

where $l_{eff,h}$ is the effective perforation length including end correction, $S_{h}=\pi D_{n}^{2}/4$ is the perforation area, and $V_{h}$ is the equivalent cavity volume associated with the Helmholtz-type path. Accordingly, the specific surface impedance of the HRSS unit can be obtained from the transfer matrix elements as

(8) $$Z_{HRSS}=A_{0}\frac{T_{11}^{\left(HRSS\right)}}{T_{21}^{\left(HRSS\right)}}$$

where $A_{0}$ is the total incident area of the HRSS unit, and $T_{11}^{\left(HRSS\right)}$ and $T_{21}^{\left(HRSS\right)}$ are the corresponding elements of $T_{HRSS}$. This formulation indicates that the HRSS unit is governed by the coupling between the slit-waveguide path and the Helmholtz-type side branch, thereby enabling multimode resonance and generating low- and high-frequency absorption peaks.

For the embedded HR unit, the acoustic behavior is described using the impedance transfer idea. The transfer matrix of the HR unit can be written as

(9) $$T_{HR}=T_{n}T_{C}$$

where $T_{n}$ and $T_{C}$ are the transfer matrices of the neck and the backing cavity, respectively. The neck matrix is given by [27]

(10) $$T_{n}=\left[\begin{array}{cc} 1 & Z_{n}\\ 0 & 1 \end{array}\right]$$

with

(11) $$Z_{n}=R_{n}+j\omega M_{n}$$

where $R_{n}$ is the neck resistance and $M_{n}$ is the neck acoustic mass. The cavity matrix can be expressed as [27]

(12) $$T_{C}=\left[\begin{array}{cc} 1 & jS_{c}Z_{0}\left(k_{0}hc\right)\\ jS_{c}\left(k_{0}hc\right)/Z_{0} & 1 \end{array}\right]$$

where $S_{c}$ is the cavity cross-sectional area, $k_{0}=\omega /c_{0}$ is the air wave number, and *hc* is the cavity height. Then, the specific surface impedance of the HR unit can be written as

(13) $$Z_{HRSS}=S_{c}\frac{T_{11}^{\left(HR\right)}}{T_{21}^{\left(HR\right)}}$$

where $T_{11}^{\left(HR\right)}$ and $T_{21}^{\left(HR\right)}$ are the corresponding elements of $T_{HR}$. This embedded HR branch mainly contributes an additional absorption peak in the middle-frequency range. The modeling route is conceptually consistent with the impedance transfer treatment of resonant cavity structures reported in the literature.

Substituting Equations (8) and (13) into Equation (2) yields the total specific surface acoustic impedance *Z_total* of the HRSS-HR composite unit cell. Finally, substituting the total impedance into Equation (1) yields the sound absorption coefficient of the composite unit cell. Through this formulation, the HRSS branch contributes low- and high-frequency absorption peaks through multimode resonance, while the embedded HR branch introduces an additional middle-frequency peak, enabling the proposed composite unit cell to exhibit multiple characteristic absorption peaks over a broad frequency range.

### 3.2. Numerical Calculation Model

To further investigate the sound absorption performance of the proposed HRSS-HR composite unit cell and validate the theoretical model, we established a three-dimensional finite element model in COMSOL Multiphysics 6.2. We performed the numerical simulation using the Pressure Acoustics and Thermo-viscous Acoustics modules. This modeling strategy is particularly suitable because thermo-viscous effects strongly influence acoustic energy dissipation in the narrow slit, perforation, and neck regions—effects that a purely lossless acoustic model cannot accurately capture. The numerical model was established according to the geometric configuration shown in Figure 3, and the corresponding parameter values are summarized in Table 1.

**Figure 5:**
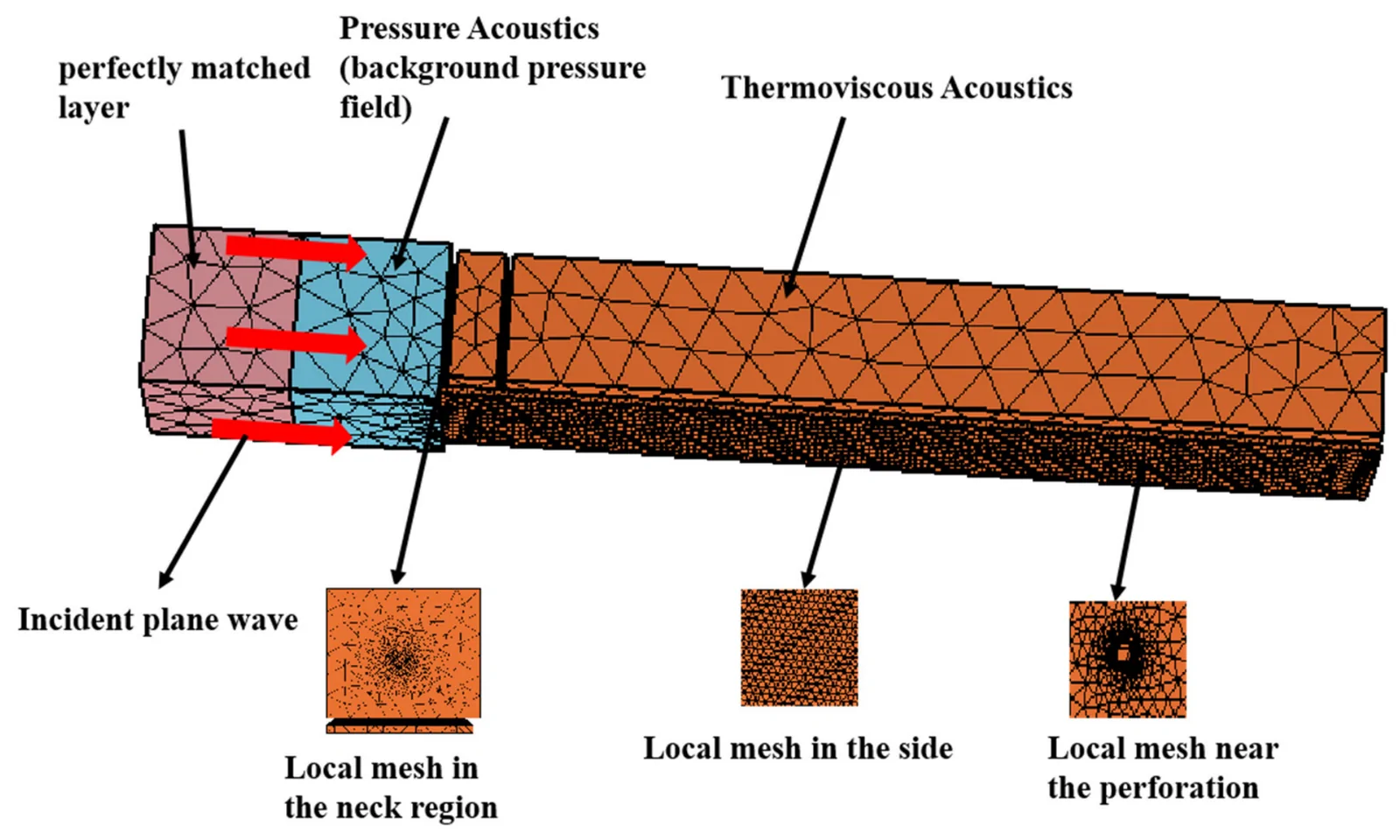
Finite element model of the HRSS-HR composite unit cell.

Figure 5 shows the finite element model of the proposed composite unit cell. In the numerical setup, the upper air domain was assigned as a perfectly matched layer (PML) to eliminate the influence of reflected waves on the simulation results. The incident acoustic field was modeled as a normally incident plane wave with an acoustic pressure amplitude of 1 Pa. Since the structure is backed by a rigid wall, the bottom boundary of the computational domain was treated as an acoustically rigid boundary.

To accurately capture the local dissipation mechanism, the side-slit region, the perforation region, and the neck region were all modeled using the Thermo-viscous Acoustics interface. By contrast, the remaining acoustic domains were modeled using the Pressure Acoustics interface. This partitioned treatment makes it possible to preserve the dominant thermal and viscous boundary-layer losses in the narrow flow passages while maintaining reasonable computational efficiency for the whole model. Similar numerical treatments have also been adopted in previous HRSS-based and resonator-coupled acoustic metamaterial studies.

Because the proposed unit cell is periodically extendable in the in-plane directions, periodic boundary conditions were imposed on the lateral surfaces of the computational domain. In addition, fine meshes and boundary-layer meshes were introduced in the slit, perforation, and neck regions to ensure sufficient numerical resolution of the thermo-viscous effects, whereas the remaining air domains were discretized using relatively coarser meshes to reduce the computational cost. Through this mesh arrangement, the numerical model can simultaneously resolve the local acoustic dissipation inside the narrow channels and the global acoustic response of the entire composite unit cell.

Based on the calculated acoustic pressure and particle velocity fields, the surface acoustic impedance of the composite unit cell was extracted, and the corresponding sound absorption coefficient was obtained using Equation (1). In addition to the absorption coefficient, the simulated acoustic field distributions were further used to analyze the underlying physical mechanisms of the proposed structure at representative resonant frequencies. In this way, the numerical model serves not only as a validation tool for the theoretical formulation, but also as an effective means to reveal the coupling effect between the HRSS branch and the embedded HR branch.

Fine meshes and boundary-layer meshes were introduced in the slit, perforation, and neck regions to ensure sufficient numerical resolution of the thermo-viscous effects, whereas the remaining air domains were discretized using relatively coarser meshes to reduce the computational cost. The computational mesh consisted of approximately 64,401 tetrahedral elements, with refined meshes in the narrow slit, perforation, and neck regions to resolve thermo-viscous boundary layers. The maximum element size in these regions was set to 0.2 mm, while a coarser mesh was used in the main cavity and air domains. Through this mesh arrangement, the numerical model can simultaneously resolve the local acoustic dissipation inside the narrow channels and the global acoustic response of the entire composite unit cell.

### 3.3. Experimental Validation and Modal Interpretation

**Figure 6:**
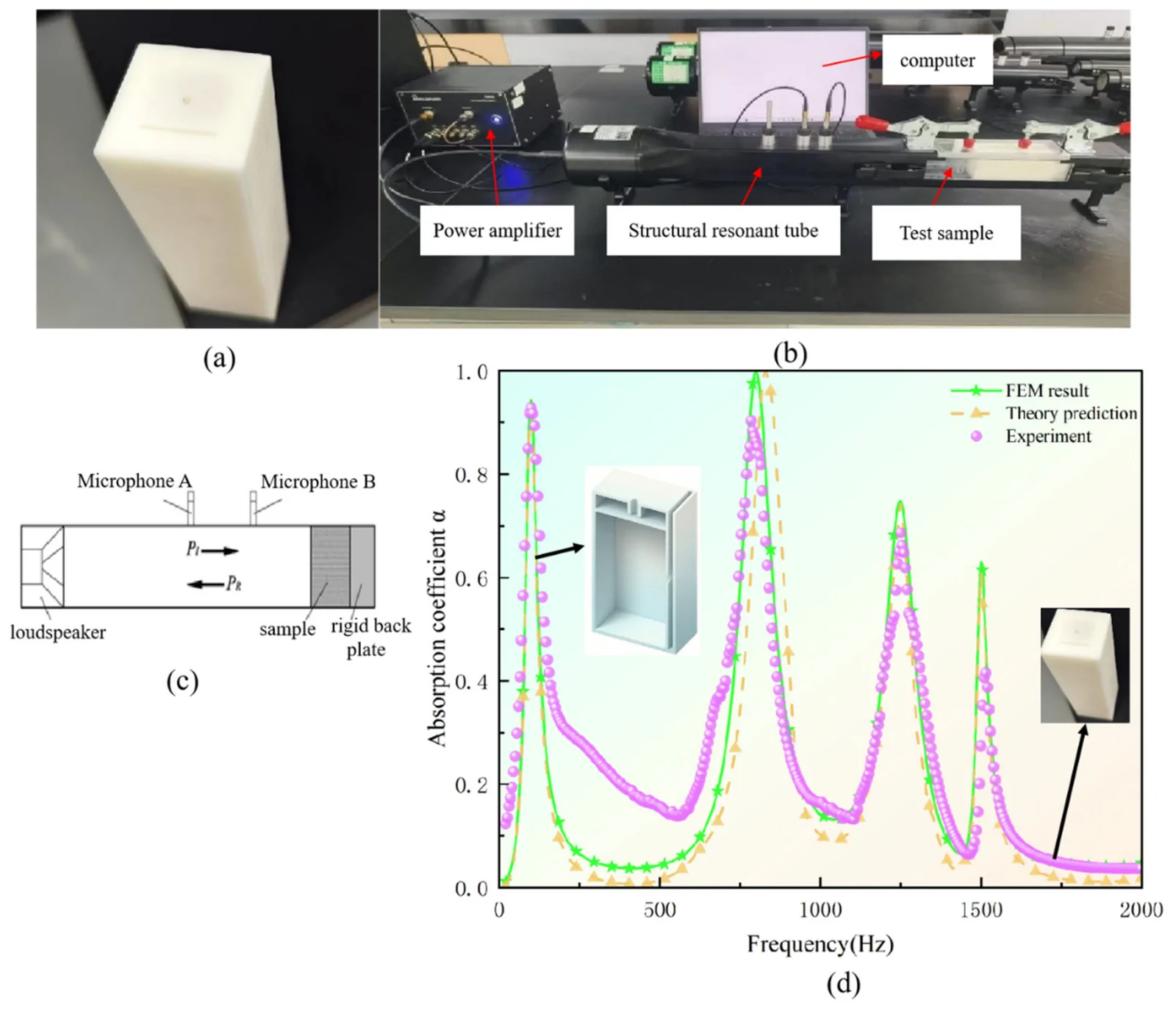
Experimental validation and acoustic performance analysis of the HRSS-HR composite unit cell showing: (**a**) the fabricated specimen; (**b**) the impedance tube setup; (**c**) the schematic of the experimental principle; (**d**) a comparison among theoretical, numerical, and experimental sound absorption coefficients.

To validate the proposed theoretical and numerical models, systematic sound absorption tests were conducted on the HRSS-HR composite unit cell using an impedance tube, as shown in Figure 6. We fabricated the test specimen by photopolymer resin 3D printing according to the geometric parameters listed in Table 1. The specimens were fabricated using stereolithography (SLA) 3D printing technology with a Formlabs Form 3+ printer. The printing material was a standard photopolymer resin (manufacturer: Anycubic, type: Standard Resin V2, Shenzhen Anycubic Technology Co., Ltd., Shenzhen, China) with a density of approximately 1.15 g/cm^3^ and an elastic modulus of approximately 1500 MPa. The printing layer thickness was set to 0.05 mm. The specimens were post-cured under UV light for 30 min to ensure complete polymerization. The environmental conditions during the experiments were maintained at room temperature (approximately 22 ± 2 °C) with a relative humidity of 45 ± 5%. It has been reported that 3D printing conditions significantly affect the quality and precision of printed samples and consequently their acoustic absorption properties. Considering that the cross section of the impedance tube is 33 mm × 33 mm, an additional outer wall thickness was introduced to match the sample size with the tube without changing the internal sound-absorbing structure, as shown in Figure 6a. The impedance tube experimental [28,29] setup is shown in Figure 6b. The experimental principle is further illustrated in Figure 6c, which provides the basis for the measurement of the normal-incidence sound absorption coefficient using the transfer-function method [30,31,32]. The measurements were performed in accordance with ISO standard [33] for the determination of the normal-incidence sound absorption coefficient using the transfer-function method. The sound absorption coefficient was calculated from the complex transfer function between two microphone positions, from which the reflection coefficient was derived. The impedance tube had a cross-section of 33 mm × 33 mm. The acquisition system comprised an NI cDAQ-9188 platform (National Instruments, Austin, TX, USA) and G.R.A.S. 40PP free-field microphones (G.R.A.S. Sound & Vibration, Holte, Denmark). The measured results were then compared with the theoretical prediction and FEM simulation to verify the reliability of the proposed HRSS-HR composite structure.

A comparison among the experimental results, theoretical prediction, and FEM simulation is shown in Figure 6d. Overall, the three results agree well in terms of the main absorption-peak positions and the overall variation trend, confirming the reliability of the theoretical and numerical models. Four characteristic absorption peaks can be observed near 100 Hz, 800 Hz, 1250 Hz, and 1500 Hz, and all of them are captured by the experimental curve, indicating that the proposed HRSS-HR composite unit cell can effectively generate multi-peak sound absorption. At the low-frequency peak around 100 Hz, the theoretical, numerical, and experimental results are highly consistent, demonstrating the good low-frequency absorption performance of the composite unit cell. However, slight frequency shifts and reductions in peak absorption coefficients can be observed at the peaks around 800 Hz, 1250 Hz, and 1500 Hz. Compared with the theoretical and FEM results, the experimental absorption coefficients at 800 Hz and 1250 Hz decrease by approximately 10%, while the reduction at 1500 Hz reaches about 25%, indicating that the higher-frequency resonance is more sensitive to practical manufacturing and installation errors. The decrease in absorption coefficient is mainly attributed to sound leakage caused by assembly gaps, while the frequency shifts are associated with dimensional deviations, surface roughness, residual material in the narrow slit and neck regions during 3D printing, and additional coupling effects induced by specimen vibration during the test. These factors are not fully considered in the ideal theoretical and numerical models. Nevertheless, the overall agreement among theory, simulation, and experiment confirms the validity of the proposed model and the feasibility of the HRSS-HR composite structure for broadband sound absorption.

**Figure 7:**
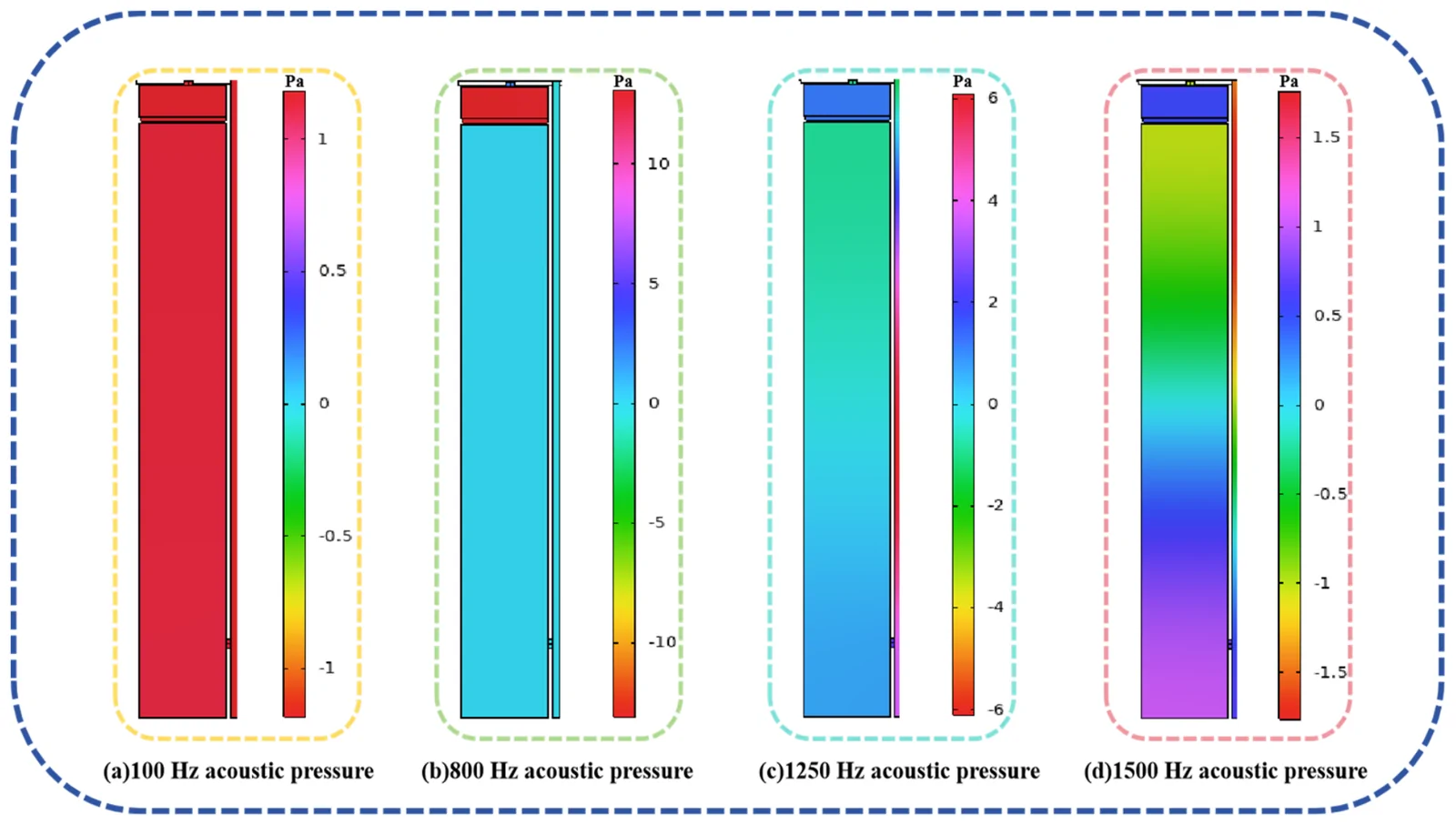
Acoustic pressure distributions at the four characteristic absorption peaks of the HRSS-HR composite unit cell.

To further clarify the physical origin of the four characteristic absorption peaks, the acoustic pressure distributions at the corresponding resonant frequencies were analyzed, as shown in Figure 7. At the first peak, the acoustic pressure is distributed almost uniformly over the main cavity, indicating a global cavity-compression mode of the composite unit cell and corresponding to a typical low-frequency Helmholtz-type resonance. At the second peak, the pressure field becomes strongly concentrated in the embedded HR unit, while the acoustic pressure in the HRSS unit is much weaker, indicating that this peak is mainly governed by the resonance of the embedded HR branch. At the third peak, the acoustic pressure is mainly concentrated in the side-slit region of the HRSS unit, revealing a slit-dominated resonance mode of the HRSS branch. At the fourth peak, the pressure field in the HRSS unit becomes more complex, with multiple alternating high- and low-pressure regions appearing in the cavity, indicating a higher-order multimode resonance caused by the coupling between the side-slit resonance and the cavity resonance.

It should be noted that the embedded HR region still exhibits a noticeable pressure response at all four peaks, which indicates strong acoustic coupling between the HR branch and the overall resonant field. However, this does not imply that all peaks are dominated by the HR branch. Instead, it reflects that the embedded HR unit actively participates in the overall resonant response of the composite cell. Overall, the first, third, and fourth peaks are mainly associated with different resonance modes of the HRSS branch, whereas the second peak is primarily governed by the embedded HR branch. These results demonstrate that the multi-peak broadband absorption behavior of the proposed composite unit cell originates from the cooperative action of the HRSS branch and the embedded HR branch.

## 4. Multi-Resonance Absorption Peaks Design

### 4.1. Parametric Study of the HRSS-HR Composite Unit Cell

To further investigate the tunability of the proposed HRSS-HR composite unit cell, we conducted a parametric study on several key geometric parameters. Specifically, the perforation depth *dl*, slit length *Lc*, slit width *t*, partition height *hc*, neck height *Ln*, and neck diameter *dn* were selected as representative parameters, and their effects on the sound absorption coefficient and characteristic absorption peaks were systematically analyzed.

All results presented in this section were obtained from FEM simulations unless otherwise stated.

**Figure 8:**
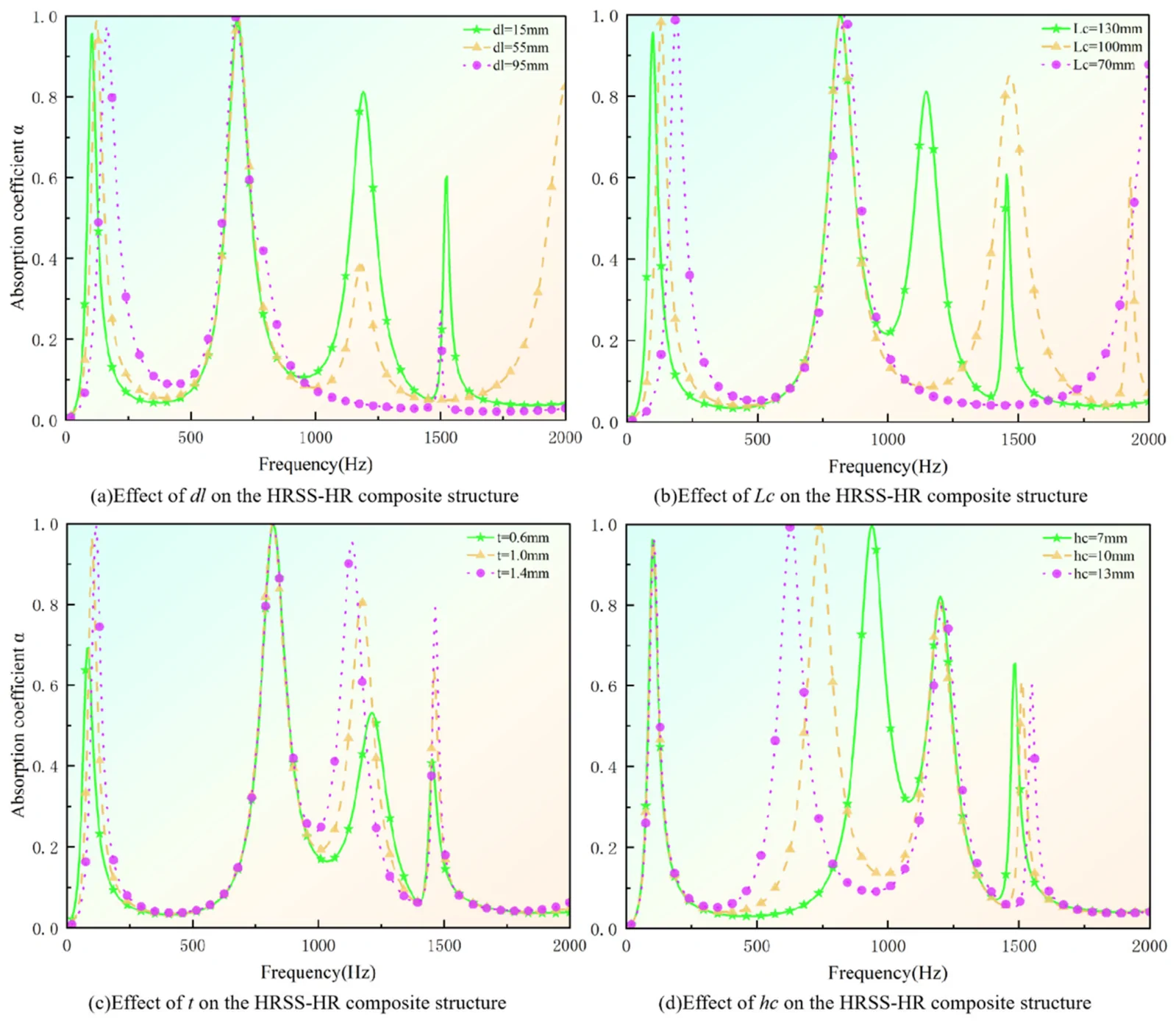

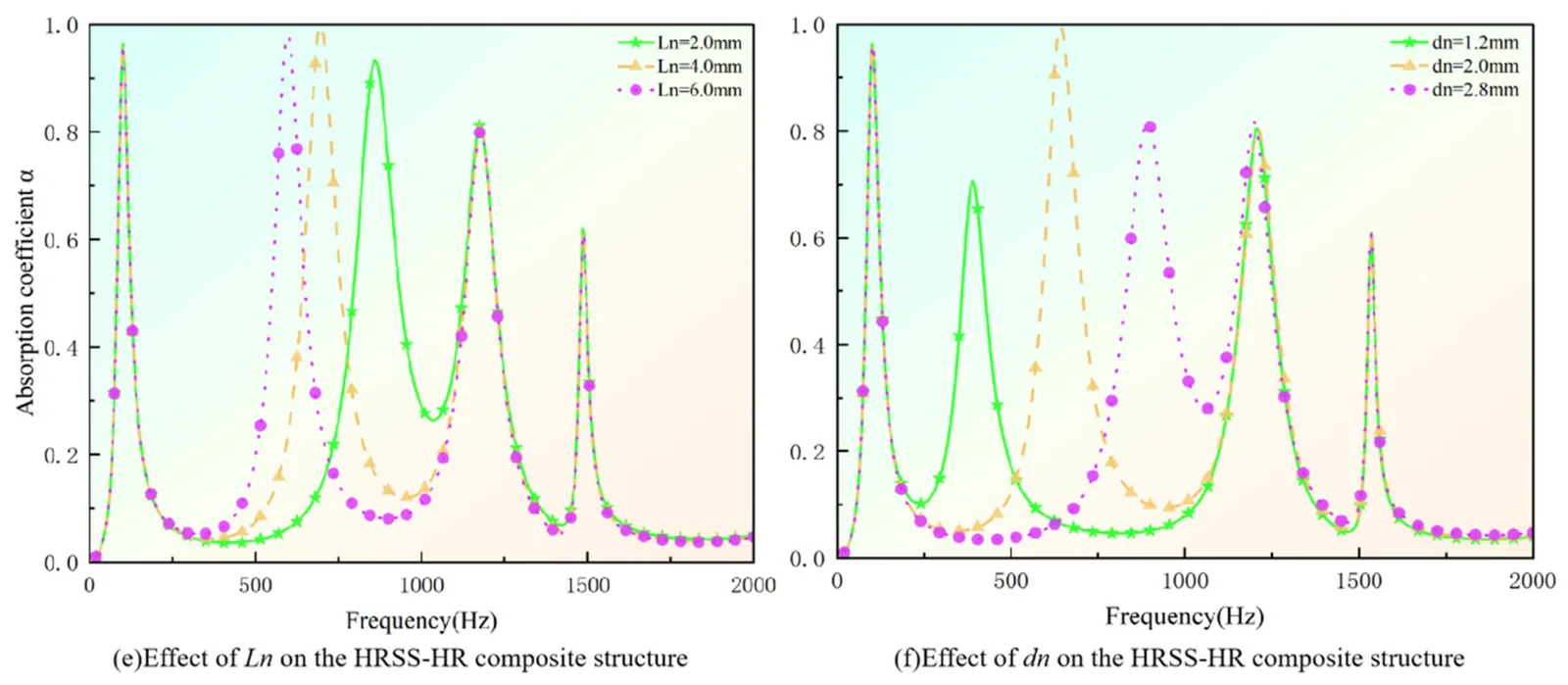
FEM-simulated parametric effects on the sound absorption performance of the HRSS-HR composite unit cell.

Figure 8a illustrates the effect of the perforation depth *dl*. As *dl* decreases, the low-frequency absorption peak shifts toward lower frequencies, and the higher-frequency peaks become more clearly separated. This indicates that the perforation depth mainly affects the coupling relationship between the Helmholtz-type resonance path and the slit-related resonance modes in the HRSS branch. Therefore, *dl* is an important parameter for promoting multimode resonance and improving the multi-peak absorption behavior of the composite unit cell.

Figure 8b shows the effect of the slit length *Lc* on the sound absorption performance. As *Lc* increases, the low-frequency peak and the higher-frequency peaks gradually shift toward lower frequencies, whereas the middle-frequency peak remains nearly unchanged. This indicates that the slit length mainly controls the effective acoustic path of the HRSS branch and therefore plays a dominant role in tuning the low- and high-frequency resonances. The relatively stable middle-frequency peak further confirms that this resonance is mainly associated with the embedded HR branch rather than the HRSS branch.

Figure 8c presents the influence of the slit width *t*. With increasing slit width, the absorption coefficients of the low-frequency and higher-frequency peaks are significantly enhanced, whereas the corresponding resonance frequencies change only slightly. This suggests that the slit width mainly affects the impedance matching condition and acoustic energy dissipation efficiency, rather than the resonance frequency itself. In other words, increasing the slit width improves the coupling efficiency between the incident sound wave and the resonant structure, thereby enhancing the overall absorption level.

Figure 8d illustrates the effect of the partition height *hc*. As *hc* decreases, the characteristic peak in the middle-frequency range gradually shifts toward higher frequencies, while the low-frequency and higher-frequency peaks remain nearly unchanged. This demonstrates that the partition height mainly regulates the resonance behavior of the embedded HR branch and provides an effective degree of freedom for selectively tuning the middle-frequency absorption peak. Such a selective tuning feature is beneficial for achieving multi-peak design without significantly disturbing the other resonances.

Figure 8e shows the effect of the neck height *Ln*. As *Ln* increases, the middle-frequency peak gradually shifts toward lower frequencies, while its peak amplitude first increases and then decreases. This indicates that the neck height mainly changes the equivalent acoustic mass of the HR branch and simultaneously affects the impedance matching condition. Therefore, an appropriate neck height is required to balance resonance frequency tuning and absorption efficiency.

Figure 8f presents the influence of the neck diameter *dn*. As *dn* increases, the middle-frequency absorption peak moves toward higher frequencies, and its peak value also shows a tendency to first increase and then decrease. This behavior suggests that the neck diameter mainly affects the equivalent acoustic mass and acoustic resistance of the HR branch. An excessively small or excessively large neck diameter weakens the matching condition between the resonator and air, whereas an intermediate value provides better absorption performance.

Overall, the parametric study shows that HRSS-related parameters govern the low- and high-frequency resonances, whereas HR-related parameters regulate the middle-frequency peak, providing a relatively decoupled tuning mechanism for broadband absorption design.

### 4.2. Absorption Recovery of the Five-Unit Parallel Structure

**Figure 9:**
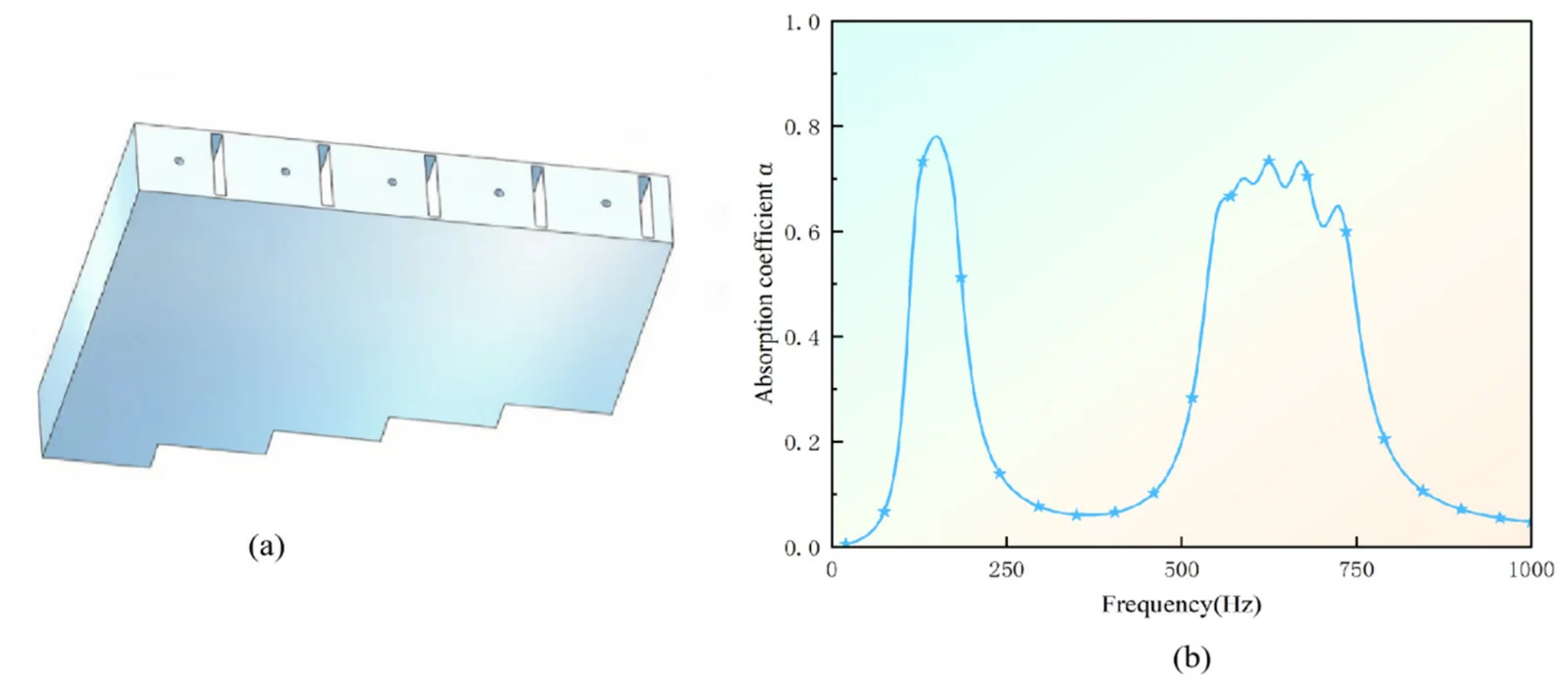
Five-unit parallel structure and its FEM-simulated sound absorption performance: (**a**) schematic of the five-unit parallel structure; (**b**) sound absorption coefficient.

To further broaden the effective absorption bandwidth, we constructed and numerically investigated a five-unit parallel structure. The results in this section were obtained from FEM simulations. As shown in Figure 9a, five HRSS-HR composite unit cells were arranged side by side on the same rigid backing, and adjacent units were separated by thin walls to maintain structural integrity and acoustic sealing. We assigned different slit lengths and partition heights to the five units to intentionally stagger their resonance frequencies, thereby expanding the overall absorption bandwidth. The corresponding geometric parameters are listed in Table 2, whereas the remaining parameters were identical to those of the optimized single unit cell.

The simulated absorption coefficient of the five-unit parallel structure is shown in Figure 9b. Compared with the optimized single unit cell, the effective absorption bandwidth is broadened after parallel coupling. However, the peak absorption coefficients in the low- and middle-frequency ranges decrease, indicating that the acoustic coupling among adjacent units weakens the original impedance matching condition. Therefore, although the parallel arrangement is beneficial for broadband absorption, the coupling effect also causes partial deterioration in the peak absorption performance.

**Figure 10:**
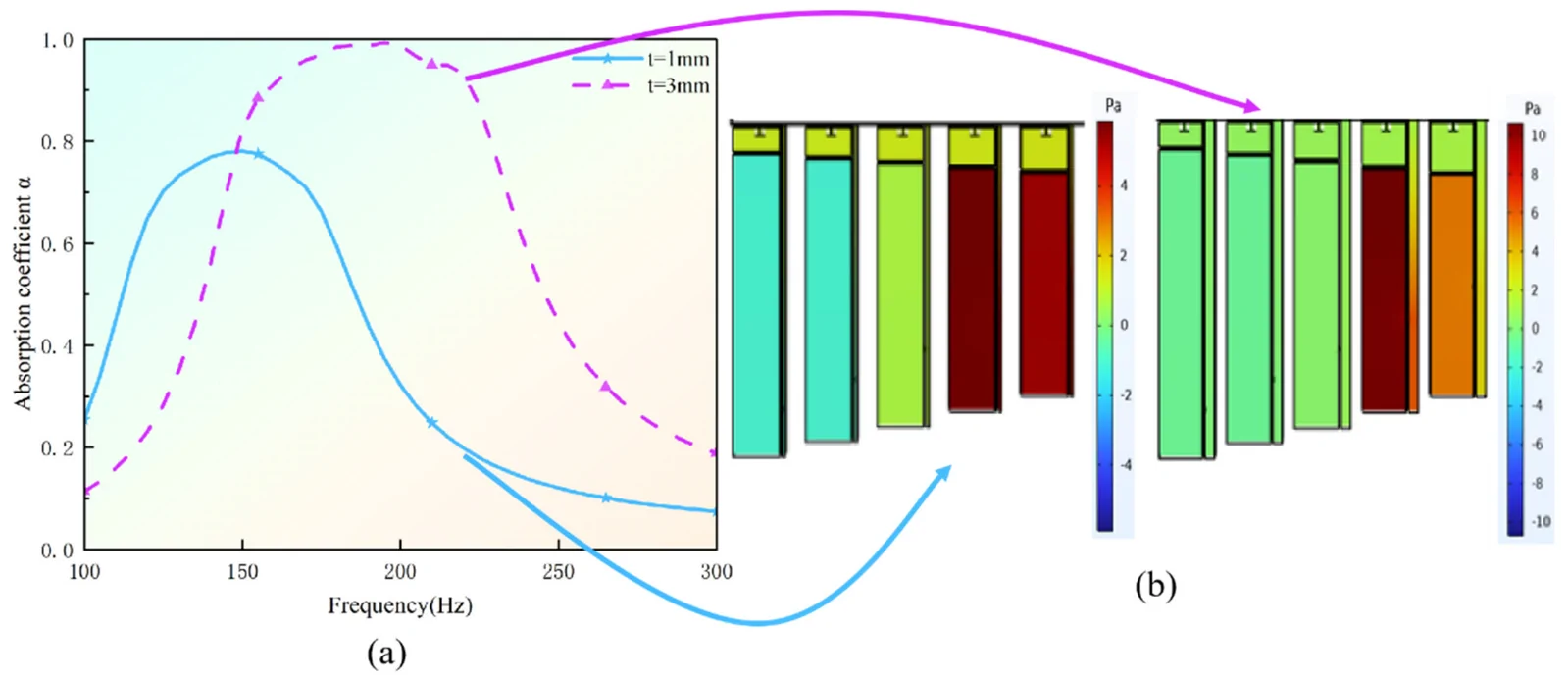
FEM-simulated recovery of low-frequency absorption by slit-width optimization: (**a**) absorption coefficient before and after optimization; (**b**) acoustic pressure distributions before and after optimization.

To recover the reduced low-frequency absorption performance, the slit width *t* was further adjusted. As shown in Figure 10a, increasing the slit width significantly restores the absorption coefficient in the low-frequency band. When the slit width was increased from 1 mm to 3 mm, the low-frequency absorption coefficient increased markedly, and the effective absorption band was also broadened. Figure 10b shows the acoustic pressure distributions at the respective absorption peaks before and after optimization (i.e., where the maximum absorption coefficients occur). After optimization, the internal acoustic pressure becomes much stronger, indicating that the low-frequency resonance is more effectively excited. This suggests that increasing the slit width mainly improves the impedance matching condition and strengthens the internal resonance of the parallel structure, thereby enhancing the low-frequency absorption performance.

**Figure 11:**
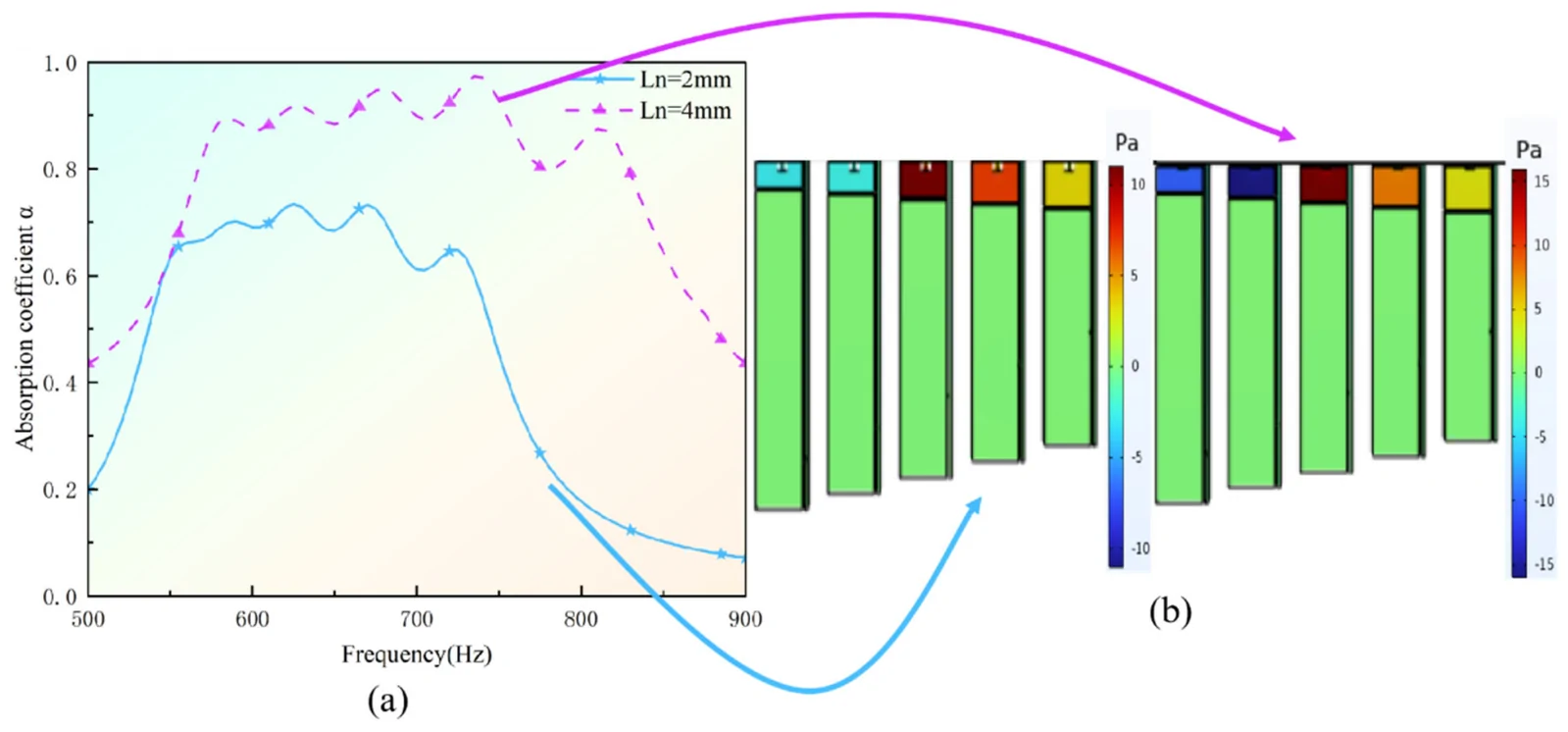
FEM-simulated recovery of middle-frequency absorption by neck-height optimization: (**a**) absorption coefficient before and after optimization; (**b**) acoustic pressure distributions before and after optimization.

To recover the middle-frequency absorption performance, the neck height *Ln* was further optimized. As shown in Figure 11a, reducing the neck height can effectively improve the absorption coefficient around the middle-frequency peak. When the neck height was decreased from 4 mm to 2 mm, the middle-frequency absorption coefficient was significantly increased, accompanied by a slight broadening of the effective absorption band. Figure 11b shows the acoustic pressure distributions at the respective middle-frequency absorption peaks before and after optimization (i.e., where the maximum absorption coefficients occur in the middle-frequency range). After optimization, the internal acoustic pressure in the resonant region is significantly enhanced, which indicates that the middle-frequency resonance of the embedded HR branch is more effectively activated. This means that decreasing the neck height mainly regulates the equivalent acoustic mass and resonance matching of the embedded HR branch under coupled conditions, thereby restoring the middle-frequency absorption performance.

Through targeted parameter regulation, the deterioration in absorption performance caused by parallel coupling can be effectively compensated. When multiple unit cells are connected in parallel, increasing the slit width is beneficial for restoring the low-frequency absorption coefficient, whereas decreasing the neck height is more effective for recovering the middle-frequency absorption performance. A summary of the regulation strategies and corresponding recovery effects is given in Table 3. These findings provide a practical basis for the subsequent design of larger array-type broadband acoustic metamaterials.

### 4.3. Structural Design and Numerical Calculation Model of the 4 × 4 Array

**Figure 12:**
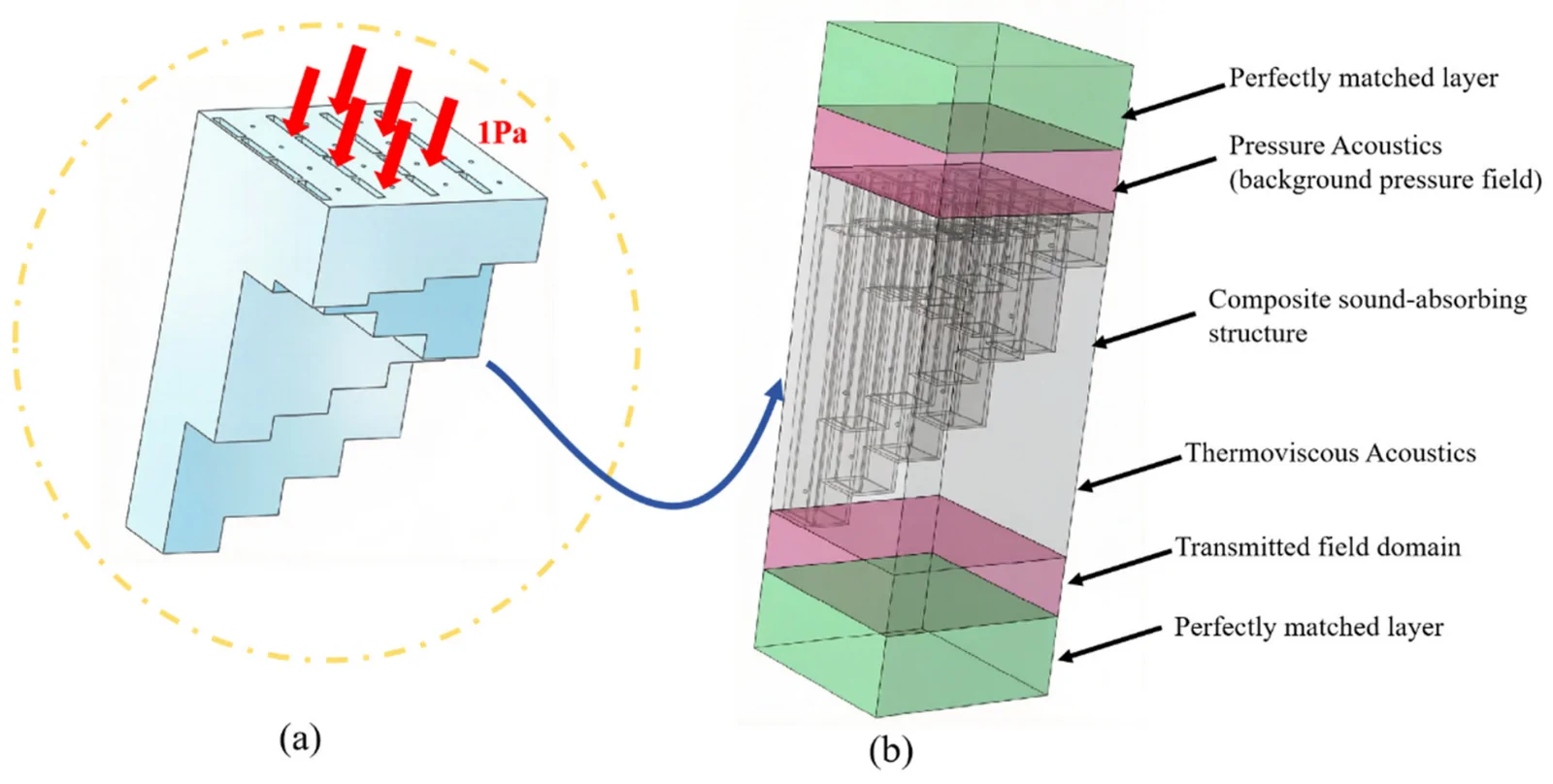
Structural configuration and FEM simulation model of the 4 × 4 HRSS-HR composite metamaterial array: (**a**) structural configuration; (**b**) finite element model of the array.

Based on the parameter regulation strategy from the five-unit parallel structure, we further designed a 4 × 4 HRSS-HR composite metamaterial array to evaluate its broadband sound absorption performance under larger-scale coupling. As shown in Figure 12a, the array consists of 16 composite unit cells arranged in a two-dimensional periodic form. In the horizontal direction, adjacent unit cells are separated by 3 mm thin walls, whereas in the vertical direction they are directly attached without any partition. To broaden the overall absorption bandwidth, the slit length *Lc* and partition height *hc* of different unit cells were designed in a staggered manner, so that the resonance frequencies of the cells were intentionally distributed over the target frequency range. Meanwhile, the slit width *t* and neck height *Ln* were uniformly selected according to the recovery trends revealed in Section 4.2, thereby providing favorable conditions for low-frequency absorption enhancement and middle-frequency resonance recovery. In the present design, the slit width was uniformly set to 4 mm, whereas the neck height was uniformly set to 1 mm for all unit cells in the array. Although the parametric study in Section 4.2 focused on a slit width up to 3 mm and a neck height down to 2 mm, a further increase in the slit width to 4 mm and reduction in the neck height to 1 mm were adopted for the 4 × 4 array to fully exploit the recovery effect. The validity of this extended parameter choice is confirmed by the subsequent simulation and experimental results (Figure 12). The remaining geometric parameters were kept the same as those of the optimized composite unit cell.

The staggered distributions of the slit length *Lc* and partition height *hc* in the 4 × 4 array can be expressed as

(14) $$Lc=\left[\begin{array}{c} \begin{array}{c} 150\\ 110 \end{array}\\ 60\\ 55 \end{array}\begin{array}{c} \begin{array}{c} \;\;140\\ \;\;100 \end{array}\\ \;\;65\\ \;\;50 \end{array}\begin{array}{c} \begin{array}{c} \;\;130\;\;\\ 90 \end{array}\\ 70\\ 45 \end{array}\begin{array}{c} \begin{array}{c} 120\\ 80 \end{array}\\ 75\\ 40 \end{array}\right]\;\mathrm{mm}$$

(15) $$\;hc=\left[\begin{array}{c} \begin{array}{c} \;29\;\;\\ 21 \end{array}\;\\ 13\\ 9 \end{array}\begin{array}{c} \begin{array}{c} 27\;\;\\ 19 \end{array}\\ 12\\ 8 \end{array}\begin{array}{c} \begin{array}{c} 25\;\;\\ 17 \end{array}\\ 11\\ 7 \end{array}\begin{array}{c} \begin{array}{c} 23\\ 15 \end{array}\\ 10\\ 6 \end{array}\right]\;\mathrm{mm}$$

where each element corresponds to the geometric parameter of the unit cell located at the same row and column position in the array. Such a differential design of *Lc* and *hc* enables the resonance peaks of different unit cells to be staggered, which is beneficial for achieving broadband sound absorption at the array level.

The remaining uniform geometric parameters are summarized in Table 4.

To investigate the acoustic response of the array, a three-dimensional finite element model was established in COMSOL Multiphysics. As shown in Figure 12b, the simulation was performed using the Pressure Acoustics and Thermo-viscous Acoustics modules. The upper air domain was assigned as a perfectly matched layer (PML) to suppress reflected-wave interference, and a normally incident plane wave with an acoustic pressure amplitude of 1 Pa was applied as the excitation source. The slit, perforation, and neck regions were modeled using the Thermo-viscous Acoustics interface to account for thermal and viscous boundary-layer losses, whereas the remaining air domains were treated using the Pressure Acoustics interface.

Fine meshes and boundary-layer meshes were introduced in the narrow slit and neck regions to ensure sufficient numerical resolution of the local dissipation mechanism, while relatively coarser meshes were used in the other regions to improve computational efficiency. Through this treatment, the finite element model can simultaneously capture the local thermo-viscous dissipation in the narrow channels and the global acoustic response of the 4 × 4 array. Therefore, the established numerical model provides an effective basis for the subsequent analysis of the broadband absorption performance of the proposed array.

### 4.4. Comparison of Simulated and Experimental Absorption Coefficients

**Figure 13:**
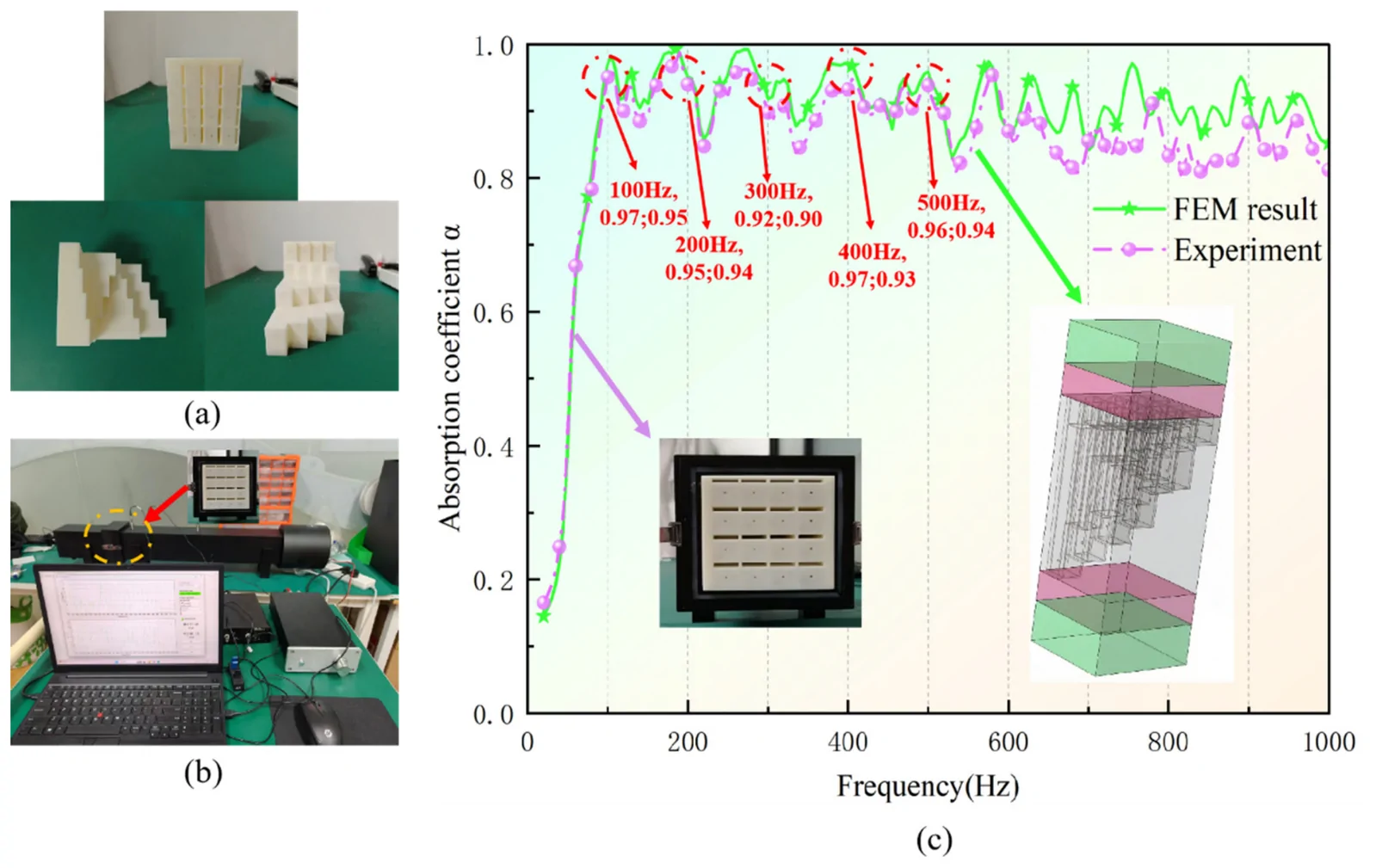
Comparison between FEM-simulated and experimentally measured sound absorption coefficients of the 4 × 4 array: (**a**) test sample; (**b**) impedance tube experiment; (**c**) comparison of simulated and measured results.

To validate the sound absorption performance of the 4 × 4 HRSS-HR composite metamaterial array, we conducted impedance tube experiments, as shown in Figure 13a,b. To satisfy the cross-sectional dimensions of the impedance tube (101.5 mm × 101.5 mm), the array specimen was thickened by introducing non-functional boundary extensions around the periphery, while keeping the internal structural parameters identical to those used in the numerical model.

The comparison between the simulated and measured sound absorption coefficients is presented in Figure 13c. It can be observed that the optimized array exhibits good broadband absorption performance within the frequency range of 100–1000 Hz. In particular, the absorption coefficient increases rapidly near 100 Hz and remains at a relatively high level across the main harmonic frequency bands, indicating that the proposed array effectively targets the dominant low-frequency components of transformer noise. Compared with the multi-peak absorption behavior of a single unit cell, the array structure effectively smooths the discrete resonance peaks and transforms them into a more stable broadband absorption response.

Overall, the experimental results agree well with the simulation results in terms of the overall trend and effective absorption bandwidth. Although slight deviations can be observed at certain frequencies, mainly in the form of reduced absorption coefficients and local fluctuations, these differences are primarily attributed to manufacturing tolerances, surface roughness, residual material in narrow channels, and minor sound leakage during sample installation. In addition, the experimental curve appears slightly smoother than the simulated result, indicating the presence of additional viscous and structural dissipation in the actual structure.

The 4 × 4 HRSS-HR composite array achieves significant low-frequency absorption near 100 Hz while maintaining stable broadband performance over the range of 100–1000 Hz. These results demonstrate that the combined strategy of staggered structural parameter design and controlled geometric optimization is effective for practical broadband sound absorption in transformer noise control applications.

## 5. Conclusions

We have proposed a composite acoustic metamaterial integrating a traditional Helmholtz resonator and a side-slit Helmholtz resonator (HRSS-HR) for broadband transformer noise control. The HRSS-HR unit cell generates four characteristic absorption peaks, among which those at 100 Hz and in the 200–500 Hz band highly match the dominant components (fundamental frequency and its low-order harmonics) of transformer noise. Parametric studies reveal that HRSS-related parameters govern the low- and high-frequency resonances, while HR-related parameters regulate the middle-frequency peak, enabling decoupled multi-peak tuning. Parallel coupling of multiple units effectively broadens the effective absorption bandwidth of the structure but impairs impedance matching; however, by increasing the slit width and reducing the neck height, the low- and middle-frequency absorption performance degraded by parallel coupling can be significantly recovered. Based on this recovery strategy, a 4 × 4 HRSS-HR array is further designed and validated. Results show that the array maintains high absorption coefficients near 100 Hz and across the dominant 200–500 Hz harmonic bands, while also exhibiting good broadband absorption performance up to 1000 Hz. This work provides a compact, tunable acoustic metamaterial solution for transformer noise control and offers a valuable reference for low-frequency harmonic noise suppression in similar power equipment.

## Figures and Tables

**Table 1:** Geometric parameters of the HRSS-HR composite unit cell.

Parameter	Symbol	Value (mm)
Cavity width	*Cw*	22
Cavity depth	*Cs*	22
Slit length	*Lc*	130
Slit width	*t*	1
wall thickness	*b*	1
Aperture diameter	*Dn*	2
Perforation depth	*dl*	15
Neck diameter	*dn*	2
Neck height	*Ln*	4
Partition height	*hc*	9

**Table 2:** Parameters of Slit Length and Partition Height for the Five-Unit Parallel Array Unit Cell.

Unit Cell	1	2	3	4	5
Slit length (mm)	120	110	100	90	80
Partition height (mm)	10	11	12	13	14

**Table 3:** Summary of parameter regulation strategies for absorption recovery of the five-unit parallel structure.

Regulation Parameter	Optimized Value	Target Frequency Range	Main Effect	Recovery Result
Slit width *t*	1 mm → 3 mm	Low-frequency band	Improves impedance matching and strengthens internal resonance	Despite a slight frequency shift, the low-frequency absorption coefficient is significantly increased to above 0.9, and the effective absorption band is broadened.
Neck height *Ln*	4 mm → 2 mm	Middle-frequency band	Adjusts equivalent acoustic mass and enhances HR resonance	Middle-frequency absorption coefficient increased from below 0.8 to above 0.8, and the effective absorption band is slightly broadened.

**Table 4:** Key uniform geometric parameters of the 4 × 4 HRSS-HR composite metamaterial array.

Parameter	Parameter Symbols	Value (mm)
Cavity width	*Cw*	22
Cavity depth	*Cs*	22
Slit width	*t*	4
Perforation diameter	*Dn*	2
Perforation depth	*dl*	15
Neck diameter	*dn*	2
Neck height	*Ln*	1

## Data Availability

The original contributions presented in this study are included in the article. Further inquiries can be directed to the corresponding author.
